# Development and validation of an updated computational model of *Streptomyces coelicolor* primary and secondary metabolism

**DOI:** 10.1186/s12864-018-4905-5

**Published:** 2018-07-04

**Authors:** Adam Amara, Eriko Takano, Rainer Breitling

**Affiliations:** Manchester Centre for Synthetic Biology of Fine and Speciality Chemicals (SYNBIOCHEM), Manchester Institute of Biotechnology, School of Chemistry, Faculty of Science and Engineering, University of Manchester, 131 Princess Street, Manchester, M1 7DN UK

**Keywords:** Genome-scale metabolic modelling, *Streptomyces*, Secondary metabolism, Omics, Synthetic biology, Natural products

## Abstract

**Background:**

*Streptomyces* species produce a vast diversity of secondary metabolites of clinical and biotechnological importance, in particular antibiotics. Recent developments in metabolic engineering, synthetic and systems biology have opened new opportunities to exploit *Streptomyces* secondary metabolism, but achieving industry-level production without time-consuming optimization has remained challenging. Genome-scale metabolic modelling has been shown to be a powerful tool to guide metabolic engineering strategies for accelerated strain optimization, and several generations of models of *Streptomyces* metabolism have been developed for this purpose.

**Results:**

Here, we present the most recent update of a genome-scale stoichiometric constraint-based model of the metabolism of *Streptomyces coelicolor*, the major model organism for the production of antibiotics in the genus. We show that the updated model enables better metabolic flux and biomass predictions and facilitates the integrative analysis of multi-omics data such as transcriptomics, proteomics and metabolomics.

**Conclusions:**

The updated model presented here provides an enhanced basis for the next generation of metabolic engineering attempts in *Streptomyces*.

**Electronic supplementary material:**

The online version of this article (10.1186/s12864-018-4905-5) contains supplementary material, which is available to authorized users.

## Background

*Streptomyces* species are usually soil-dwelling bacteria, which have adapted to their competitive ecological niches by developing a notably diverse secondary metabolism (e.g., antimicrobials). Currently, more than two thirds of the antibiotics used have been derived from natural products discovered in *Streptomyces* and related species [1]; however, the antibiotic discovery pipeline is drying up, while the antimicrobial resistance threat is growing. *Streptomyces coelicolor* A3(2) is a well-studied model organism for the production of antibiotics in this genus. The genome of this soil-dwelling bacterium encodes more than 20 secondary metabolite biosynthetic gene clusters (BGCs) [2], and the species is known to produce multiple antibiotics such as Actinorhodin (Act), Undecylprodigiosin (Red), Calcium-Dependant Antibiotic (CDA) and the yellow Coelicolor Polyketide, Coelimycin P1 (yCPK) [3]. Recent developments in metabolic engineering, synthetic and systems biology have opened new opportunities to exploit *Streptomyces*’ secondary metabolism diversity to discover novel antibiotics and natural product-derived drugs [4, 5]. However, expensive and time-consuming strain optimization is usually required to achieve industrially competitive production levels. A major issue faced in strain design is the ability to integrate test data (e.g. metabolomics) to improve the design [6], and many of the issues encountered are related to metabolic optimization, such as metabolic bottlenecks to increase production [7], heterologous biosynthetic pathway precursors production [8], or accurate predictions for metabolic engineering [9].

Genome-scale metabolic models (GSMM) have been shown to be a powerful tool to guide metabolic engineering strategies for accelerated strain optimization [10–12], and several generations of models of *Streptomyces* metabolism have been developed for this purpose [13–17]. The use of constraint-based modelling, in particular with flux balance analysis (FBA), enables the reconstruction and analysis of large metabolic networks from the genome sequence as well as predictions of growth associated phenotypes (metabolic fluxes, growth rates, metabolic gene essentiality) [18]. Informative models for this purpose can be constructed even when enzyme kinetic data or metabolite concentrations are unknown in the target organism, making this approach particularly attractive for less well-studied organisms like *Streptomyces* strains. In 2005, the first generation GSMM of *S. coelicolor*, iIB711, was published [19], which was used to identify metabolic gene knock-outs to drive the enhanced production of antibiotics in the strain [20]. In 2010, an updated model, iMA789, was published [21], which introduced more detailed antibiotics metabolic pathways and was used to interpret time-course gene expression data, which was then used to improve the model and update the genome annotation of the organism in the area of secondary metabolism. The most recent model update, iMK1208, was published by Kim et al. [22]; this model significantly expanded the number of reactions and genes, as well as updating the biomass reaction. This model was then used in a transcriptomics-based optimization for actinorhodin overproduction in *S. coelicolor* [15].

Furthermore, several genome-scale metabolic models for other biotechnologically relevant *Streptomyces* strains have been reconstructed since the first *S. coelicolor* model, iIB711. A model of the *Streptomyces tenebrarius* metabolic network, which was derived from the iIB711 model of Borodina, Krabben & Nielsen has been used to identify targets to optimize production of tobramycin [23]. A model of *Saccharopolyspora erythraea* has been reconstructed based on the iMA789 model of Alam et al. to improve the production of erythromycin [24]. One of the most recent model reconstructions derived from Kim et al.’s *S. coelicolor* iMK1208 model was used for model-guided engineering of ethylmalonyl-CoA pathways in *Streptomyces hygroscopicus* to increase production of ascomycin [14]. A large collection of minimally curated metabolic models of different *Streptomyces* strains and other actinomycetes was used to evaluate potential host strains for overproducing different chemical classes of secondary metabolites using comparative multi-objective modelling [25].

Based on recent advances in our understanding of *Streptomyces* metabolism and technical progress in the concepts of computational model building, we constructed and validated an updated GSMM of *S. coelicolor,* iAA1259, to provide a more precise metabolic flux and biomass predictions and to facilitate the integration of metabolomics, proteomics, and transcriptomics information with the model predictions.

## Results & Discussion

### Genome-scale reconstruction and characteristics updated

The construction of the updated GSMM of *S. coelicolor* A3(2), iAA1259, was based on all three previously published iterative reconstructions of *S. coelicolor* metabolic models [19, 21, 22], by updating and adding data in the model based on new genetic (e.g., gene–protein–reaction relationships) or biochemical knowledge. A summary of the main updates and new features added is available in the Additional file 1: Tables S3, S4, S5 and S6.

Multiple pathways were added or updated. 1) Polysaccharide degradation pathways (e.g., for xylan, cellulose) were introduced to enable simulated growth in complex media containing these carbon sources. 2) The biosynthetic pathway for the secondary metabolite yCPK [26–28] was added to the model. This cryptic BGC is awakened under phosphate-limited condition, in nitrogen and carbon rich media [26, 29], such as in the minimal media used for systems biology studies of *S. coelicolor* [30]. 3) The biosynthetic pathways for the signalling molecules gamma-butyrolactones (SCB1, 2 and 3) were added [31, 32]; secondary metabolite production in *Streptomyces* (e.g., yCPK) can be activated through these small diffusible molecules, and they are an interesting target for synthetic biology engineering [33, 34]. 4) The futalosine pathway, an alternative menaquinone biosynthesis pathway, which was highlighted as incomplete in the previous model [22], has now been updated following recently published studies [35, 36]. 5) The oxidative phosphorylation associated reactions have been manually curated. 6) Following the above modifications, the biomass reaction has also been updated to reflect more detailed knowledge on biomass composition such as the presence of 2-demethylmenaquinol in *S. coelicolor* (MetaCyc) [37], and organic polyphosphate storage [38], as well as an update in the stoichiometry of menaquinol based on *Mycobacterium tuberculosis* data [39] (see details in Additional file 2: Table S1).

In order to facilitate metabolomics data analysis, all metabolites in the model have now been annotated with standard identifiers for a variety of relevant databases (PubChem and ChEBI) [40, 41], and chemical and structural information about each metabolite has been added (InChi and SMILES strings) to ensure unambiguous metabolite identification [42, 43]. The model capacity to facilitate metabolomics data analysis has been tested by mapping metabolites annotated with mzMatch [44] from an untargeted metabolomics dataset of *S. coelicolor* [45]; the metabolites were mapped automatically onto the iAA1259 metabolic network (see details in Additional file 1: Figure S1). In addition, to facilitate transcriptomics data analysis and comparative modelling, gene annotation has been expanded to include identifiers for multiple standard databases (Gene Ontology, Ensembl, and RefSeq) [46–48]. Finally, to integrate proteomics data analysis, standard database identifiers (UniProt, Pfam, and Panther) [49–51] and key reference data, such as protein sequence, length, and mass, have been added. The final model, iAA1259, is fully compliant with the current standards for high-quality GSMMs [52–54], iAA1259 is available as a SBML file in Additional file 3 and as an excel file in Additional file 4.

### Validations of the metabolic model predictions

**Fig. 1:**
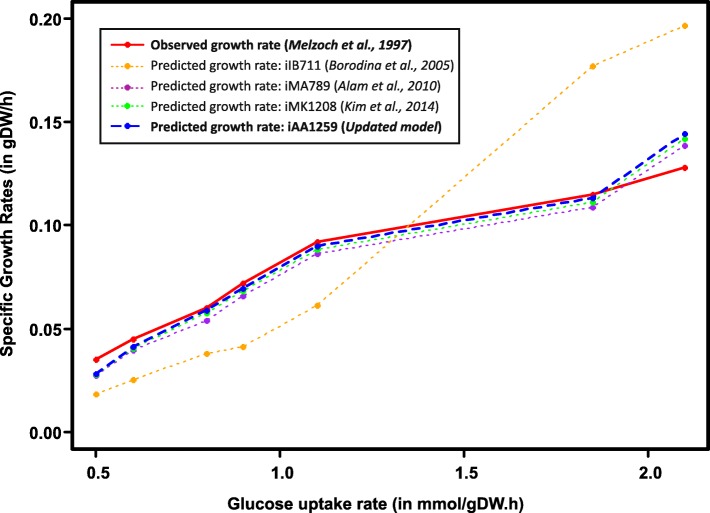
Initial model growth predictions validation. Comparison of the specific growth rate predicted *in silico* with different models to the measured growth rates in chemostat data [55] with a glucose-limited minimal defined media. The published data on the rate of glucose uptake, oxygen consumption, CO_2_ production and γ-actinorhodin production for seven different conditions were used as metabolic constraints in the different models. Growth prediction by iAA1259 shows a slight improvement compared to its immediate predecessor, iMK1208 [22]

As the first step in model validation, chemostat data collected by Melzoch et al. *for S. coelicolor* in a glucose-limited minimal defined media [55] were used to compare biomass predictions by the four generations of model: iIB711, iMA789, iMK1208, and iAA1259. Specific growth rates for each model were predicted in silico, using the known glucose and O_2_ uptake rates as constraints on the model, along with the production rates of CO_2_ and γ-actinorhodin, the extracellular lactone form of actinorhodin [56]. Biomass production was maximized to estimate the optimal predicted growth rate. Then, the growth rate predicted in silico was compared to the dilution rate that corresponds to the observed growth rate at steady state (Fig. 1). Since the first published model iIB711, there have been some significant improvements in biomass predictions; iAA1259 shows a slight improvement in predictions compared to the previous model update, iMK1208 (8.2% average error for iMK1208 predictions versus 7.0% with iAA1259). This first validation confirms that the predictive performances of the updated model iAA1259 are at least as good as the previous models generations. However, the next validation step requires more complex and quantitative datasets. The data used as constraints and the predicted growth rates data for the different models are available in Additional file 1: Table S7.

**Fig. 2:**
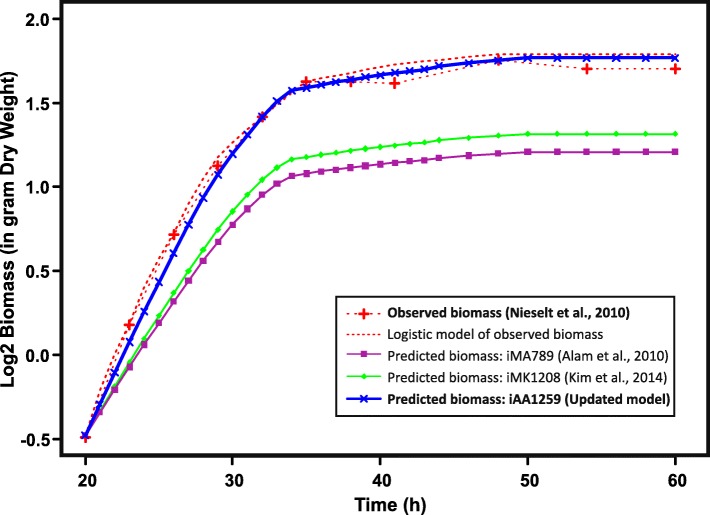
Comparison of dynamic cell growth predictions. The quantitative in silico growth predictions are compared to measured biomass and predictions with previously published models across time points. The models were constrained using phosphate, glucose, and glutamate consumption, as well as production of the antibiotics actinorhodin and undecylprodigiosin measured in a fermenter experiment [57]. The updated model’s predictions are closer to experimental observations than those of previous models, showing a significant improvement in growth prediction with the iAA1259 model

A more substantial improvement in prediction quality is observed when comparing the dynamic growth predictions of the metabolic models iAA1259, iMK1208, and iMA789, to published experimental growth data (Fig. 2) [57]. The dynamic growth was predicted by applying dynamic constraints from fermenter data (see Methods for details). The comparison of the predicted and experimental dynamic cell growth shows a significant improvement in quantitative and qualitative biomass prediction using the updated model iAA1259 (moving from an average absolute error of 37.6% with iMK1208 predictions to 5.3% with iAA1259; Fig. 2, and Additional file 1: Figure S2). This improvement in biomass predictions is most likely due to the update of the biomass reaction and in the oxidative phosphorylation-related reactions updates (i.e., cytochrome oxidases and/or menaquinone pathway), as these are the main adjustments affecting biomass-related reactions directly.

**Fig. 3:**
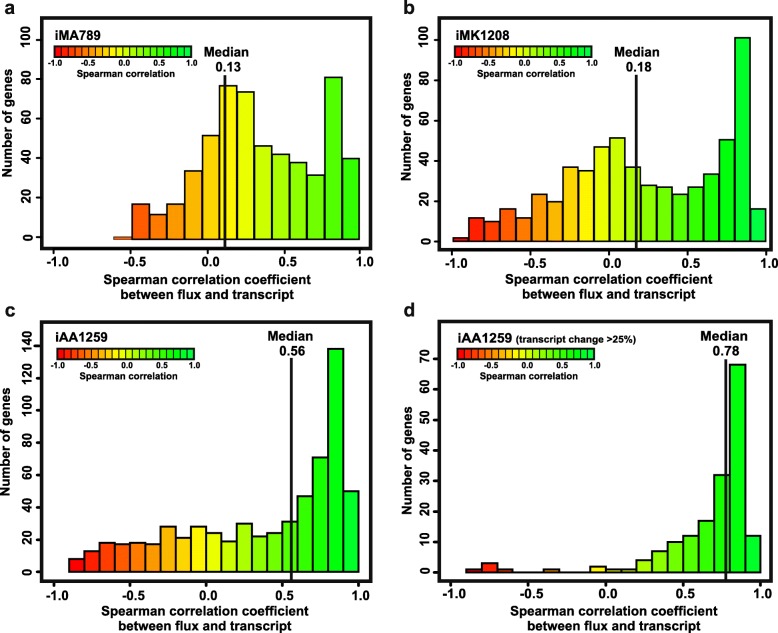
Correlation analysis between gene expression and predicted fluxes for the different models. The histograms show correlations between gene expression and flux predicted for the metabolic genes present in the different published GSMMs of *S. coelicolor*. This approach has been used first by Alam et al. [21], for the model iMA789. **a** Histogram of correlations for the model iMA789 by Alam et al. [21]. **b** Histogram of correlations for the model iMK1208 by Kim et al. [22]. **c** Histogram of correlations for the new model, iAA1259. The histogram shows a strong correlation between gene expression and predicted fluxes for metabolic genes present in the model iAA1259. Overall correlation is substantially higher than for the previous models, with a median Spearman correlation of 0.56 compared to 0.13 for iMA789 and 0.18 for iMK1208. **d** Histogram of correlations for the model iAA1259, but only taking into account genes with expression variation of more than 25% between the minimal and maximal transcript level

The next validation step involved individual metabolic flux predictions across the model. For this purpose, the models were constrained with time series fermenter data for glucose and O_2_ uptake rates and the production rates of γ-actinorhodin and CO_2_ for 32 time points from Nieselt et al. using the method introduced by Alam et al. [21]. The metabolic flux predictions were compared to time series of gene-and protein expression reported by Lahtvee and colleagues [58], as proxies for the relative metabolic flux across the time course. For the majority of genes, the gene expression changes over time are strongly correlated to the predicted metabolic fluxes through the associated reactions (Fig. 3), and the correlation is substantially improved in the updated model presented here (median Spearman correlation coefficient 0.56, compared to 0.18 in the most recent predecessor, iMK1208). When focusing only on the correlation for genes that change at least 25% in expression across the time course (Fig. 3d), i.e. those genes that should show correlation, the quality of the correlation is even more pronounced (the Pearson correlation coefficient increases by 39% from 0.56 to 0.78), and it becomes clear that only a very small number of genes show anti-correlated behaviour, i.e. a strong disagreement between gene expression and predicted fluxes. A similar trend is observed when applied to the fluxes predicted with iMA789 and iMK1208 models, both showing an increase of overall Pearson correlation from 0.13 to 0.38, and from 0.18 to 0.56, respectively (Additional file 1: Figure S1). The trend of a progressive increase in predictive power is still observed from iMA789 to iMK1208 (47% increase in correlation), and from iMK1208 to iAA1259 (42% increase in correlation).

**Fig. 4:**
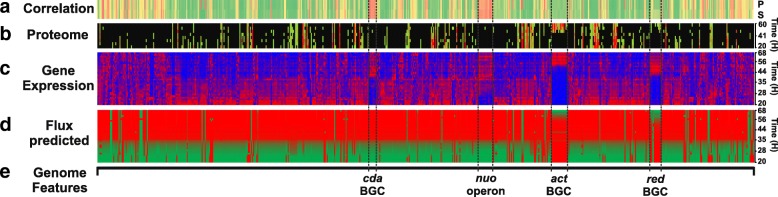
Validation by integrated transcriptomics and proteomics analysis. Gene expression and proteomics data were mapped to metabolic enzyme-coding genes and the associated metabolic fluxes predicted over time. Overall, the predicted flux trends are strongly correlated (green colour in the top bar) to the observed gene expression trend across the metabolic switch event (around between 35 and 36 h). Two highly anti-correlated gene clusters are highlighted (red colour in the top bar). **a** Correlation: Pearson (P) and Spearman (S) correlation coefficient between the experimental gene expression level and the predicted fluxes through the corresponding reaction for each individual metabolic gene (green: good correlation; yellow: no correlation; red: anti-correlation. **b** Proteome: Protein abundance observed in experimental time course data: red: high: green: low abundance, black: missing data (only a small subset of enzymes was quantified). Proteomics data from Thomas, et al. [60]. **c** Gene expression: Gene expression levels observed in the same experimental time course (red: high, blue: low expression). A much larger number of time course were studied than in the proteomics analysis. Gene expression data from Nieselt et al. [57]. **d** Predicted flux: Flux predicted during a simulated time course (green: high; red: low predicted flux). **e** Genome features: Selected genomic regions discussed in the text are annotated. The data is ordered based on the position of analysed genes in the reference genome (from left to right, from 161,237 bp to 8,468,158 bp). Genome sequence from Bentley et al. [2]

In the updated model, there are two major genetic features showing anti-correlation (Fig. 4, row a), the genes associated to Calcium Dependant Antibiotics (CDA) biosynthesis, and the *nuo* operon genes associated to an NADH dehydrogenase (complex I). The CDA genes (Fig. 4, row e) are anti-correlated because their gene expression unexpectedly increases during the transition phase (Fig. 4, row c), whereas the model does not produce CDA. The metabolite production was not switched on in the model, as there is no calcium in the media conditions used by Nieselt et al. [57]. It has been shown previously that CDA could not be detected at significant levels if there was no calcium in the media [59]. Furthermore, production of the associated proteins is not confirmed by the proteomics data [60] (Fig. 4, row b). Thus, in this case, the model prediction (no flux increase during the transition phase, Fig. 4 row d) appears to be correct, and gene expression in this exceptional case might not be correlating with metabolic flux. The Fig. 4 is available in high-definition as Additional file 5.

Regarding the second major anti-correlation, which is seen for the genes encoding the 14 subunits of NADH dehydrogenase I (NDH-I), the nuo complex, the disagreement between gene expression and predicted flux is due to a regulatory phenomenon, which in general is difficult to capture in a constraint-based model: two isoenzyme complexes are present in *S. coelicolor*, the relative expression of which is controlled by a regulatory loop dependent on NADH/NAD^+^ ratio [61]. Of these, the *nuo* genes are preferentially expressed during fumarate respiration (stationary phase, [62]). So, while fluxes through the reaction catalysed by the NADH-dehydrogenase are reduced after the transition phase, *nuo* gene expression increases and results in an anti-correlation of *nuo* gene expression with the flux prediction (which does not distinguish between the isoenzyme complexes). The second NADH dehydrogenase, NDH-II, encoded by three copies of the *ndh* gene, is preferentially expressed during exponential phase and switched off after the transition phase; hence, the *ndh* genes show high correlation with the predicted flux. While regulatory phenomena like this are not considered in this type of model, the misprediction highlights an interesting phenomenon for future study, i.e. the impact of the relative role of the two sources of reducing co-factors on secondary metabolism in *S. coelicolor*.

## Conclusions

Here, we have presented an updated computational model of *S. coelicolor* primary and secondary metabolism, iAA1259; this model shows improved predictive abilities compared to previous model generations for metabolic changes at different scales, from overall biomass dynamics to fluxes through individual reactions.

Another important improvement is that the model has been also updated to enable integrative multi-omics data analysis, to be used for designing and debugging of engineered *Streptomyces* strains using a synthetic biology approach [6], and is now fully compliant with current modelling standards [52, 54].

The model presented here will be a good basis for the next round of computer-aided design of metabolically enhanced *Streptomyces* strains. The principled construction of the model using standard identifiers will facilitate the transfer of information to related strains beyond *S. coelicolor* (e.g., recently emerging popular biotechnological hosts, such as *Streptomyces albus* and *Streptomyces venezuelae* [63, 64]). It will also serve as a solid starting point for the next generation of updated metabolic models, which will address the challenge of including kinetic and regulatory constraints, in a similar way as the recently published genome-scale metabolic models for the well-studied microorganisms *Escherichia coli* [65] and *Saccharomyces cerevisiae* [66].

## Methods

### Metabolic model reconstruction

The model reconstruction was initiated by updating the iMK1208 *S. coelicolor* model. The standard protocol for reconstruction of high-quality constraint-based GSMMs was followed when adding new genes, reactions, and metabolites [53].

In summary, the initial stoichiometric matrix was generated by comparing and using the iMK1208 model [22], KEGG [67], ScoCyc [37], and two automated reconstructions using RAST annotations and SEED reconstructions [68, 69]. The resulting matrix was manually curated for specific pathways (e.g., secondary metabolites biosynthesis, oxidative phosphorylation), to add or correct missing reactions, metabolites, genes associated, or reversibility constraints; this was supported by extensive literature survey to identify new knowledge or gaps in the previous model. Comparative analysis of transcriptomics data with iMK1208 helped to identify gene mis-annotations to be corrected [21, 57]. The biomass reaction was updated, as multiple reactions impacting biomass have been added (e.g., demethylmenaquinone, cytochrome oxidases or NADH dehydrogenase reactions). This was followed by a recalculation of the ATP fluxes for growth-associated and non-growth-associated maintenance using chemostat data [55], following the Varma & Palsson protocol [70]; the resulting values were very similar to those used in iMK1208 (with a GAM of 75.7 ATP in iMK1208 versus 75.79 ATP in iAA1259, and an NGAM of 2.65 in iMK1208 versus 2.64 in iAA1259). The detailed modifications on the biomass are available in the Additional file 2: Table S1.

Finally, multiple database identifiers were added either by automatic matching or by manual curation when necessary. The metabolites were annotated with multiple database identifiers; BiGG and KEGG identifications were already present in iMK1208, and other databases relevant to metabolomics data analysis were added: ChEBI, HMDB, CAS, IUPAC, ChemSpider, Metlin and PubChem identifications, wherever available. Chemical structure-related annotations (SMILES or InChi) were also introduced for all metabolites. Furthermore, when available, all reactions were annotated with EC code and CAS registry number, in addition to the BiGG annotation used in iMK1208. Additional gene annotations have been included to facilitate transcriptomics data integration with identifiers for Gene Ontology (GO), RefSeq, EMBL-ENA and Ensembl. For integrated proteomics analysis, annotations have been expanded to include identifiers for UniProt, Pfam and Panther, as well as data on protein length, mass, and amino acid sequence to support the direct mapping of mass-spectrometry-based proteomics data in the future. The expansion of these annotations also aims at helping fast reconstruction of metabolic models for other *Streptomyces* strains using comparative reconstruction and modelling methods. The final model has been named iAA1259 and is compliant with current metabolic model standards [52, 54]. The final model is available in SBML format and Excel format in Additional files 3 and 4.

### Constraint-based modelling

The model was analysed by using Flux Balance Analysis (FBA) and parsimonious FBA (pFBA) to predict optimal in silico growth and metabolic flux using the COBRA toolbox in Matlab and Python [71, 72] and further evaluated using OptFlux and Sybil [73, 74]. To apply condition-specific constraints corresponding to the media composition, the uptake fluxes for exometabolites not available in the medium were set to zero, while all metabolic by-products were always allowed to leave the metabolic system. The measured nutrient uptake rates from the fermenter datasets are used to define constraints of the nutrient uptake for the model. The objective function maximized in the modelling was the growth rate (steady-state flux towards biomass).

Despite the fact that FBA is not a dynamic modelling approach (its basic assumption being a steady-state flux distribution), using dynamic constraints on CO_2_, O_2_, glucose, phosphate, and glutamate uptake based on fermenter time-course data [57] enabled simulation of the growth and metabolic dynamics across time. In order to simulate the production of the main antibiotics, the biomass composition was varied dynamically depending on the observed concentration of γ-Act and Red secondary metabolites in the cultures [21].

### Transcriptomics and proteomics data analysis

Multiple omics data types have been used to validate the model; the proteomics data [60] have been acquired from the same time-series experiment samples as the flux constraints data and the transcriptomics data [57]. The transcriptomics and proteomics data were matched to corresponding metabolic genes associated with reactions by matching the StrepDB gene annotations. The matching procedure was similar to the one used for iMA789 [21]. Gene expression levels and predicted fluxes were compared using Pearson and Spearman correlations. The data used are available in the Additional file 6: Table S2.

## Additional files

Additional file 1: Containing the **Table S3.** Summary table of the updates and new features added to the iAA1259 model compared to the previous generations. **Table S4.** Table of the new reactions added to the iAA1259 model. **Table S5.** Table of the new metabolites added to the iAA1259 model. **Table S6.** Table of the new genes added to the iAA1259 model. **Figure S1*.*** Correlation analysis between gene expression and predicted fluxes for iMA789 and iMK1208 (gene expression showing a variation superior to 25%). **Figure S2**. Mapping of observed metabolites in an untargeted metabolomics dataset onto the metabolic network**. Table S7.** Constraints used and predicted growth rates of the different models from the Fig. 1**. Figure S3.** Comparison of the normalized growth prediction of the metabolic models to the experimental data. (DOC 4121 kb)

Additional file 2: **Table S1**. Biomass modifications and recalculation of ATP consumption. (XLS 81 kb)

Additional file 3: iAA1259 metabolic model in SBML format. (XML 4249 kb)

Additional file 4: Excel file specifying metabolites, reactions, genes contained, and databases IDs present in the iAA1259 metabolic model. (XLS 2531 kb)

Additional file 5 High-resolution version of Fig. 4. Validation by integrated transcriptomics and proteomics analysis. (PDF 1881 kb)

Additional file 6 **Table S2.** Full detailed data used for the Fig. 4. (XLS 1690 kb)

## Abbreviations

Act: Actinorhodin
BGC: Biosynthetic Gene Clusters
CDA: Calcium-Dependant Antibiotic
COBRA: COnstraint-Based Reconstruction and Analysis
FBA: Flux Balance Analysis
GSMM: Genome-Scale Metabolic Model
pFBA: Parsimonious Flux Balance Analysis
Red: Undecylprodigiosin
yCPK: Yellow Coelicolor Polyketide/Coelimycin P1
