# Supplementary material for: Development and validation of an updated computational model of Streptomyces coelicolor primary and secondary metabolism
Source: BMC Genomics. 2018 Jul 4;19:519. doi: 10.1186/s12864-018-4905-5 (PMC6040156; doi:10.1186/s12864-018-4905-5)
Supplement: Supplementary file 1 — Containing the Table S3. Summary table of the updates and new features added to the iAA1259 model compared to the previous generations. Table S4. Table of the new reactions added to the iAA1259 model. Table S5. Table of the new metabolites added to the iAA1259 model. Table S6. Table of the new genes added to the iAA1259 model. Figure S1. Correlation analysis between gene expression and predicted fluxes for iMA789 and iMK1208 (gene expression showing a variation superior to 25%). Figure S2. Mapping of observed metabolites in an untargeted metabolomics dataset onto the metabolic network. Table S7. Constraints used and predicted growth rates of the different models from the Fig. 1. Figure S3. Comparison of the normalized growth prediction of the metabolic models to the experimental data. (DOC 4121 kb) [file 12864_2018_4905_MOESM1_ESM.doc]

**Additional file 5**

1. **Summary of the main updates and new features present in the iAA1259 model compared to previous generations**

Table S3. Summary table of the updates and new features added to the iAA1259 model compared to the previous generations. This table shows the main updates done on the iAA1259 model compared to previous models, in particular the updates on metabolic pathways.

| **Metabolic models** | **iAA1259** |
| --- | --- |
| **Number of genes included** | 1259  (18 genes replaced + 51 new genes) |
| **Reactions** | 1912  (68 reactions modified + 53 new reactions) |
| **Metabolites** | 1470  (34 metabolites added) |
| **Additional gene databases identifiers included** | Uniprot, Gene Ontology, RefSeq, BioCyc, Pfam, PANTHER  (Already in the previous model: StrepDB) |
| **Additional metabolites databases identifiers** | Chebi, HMDB, IUPAC, CAS, SMILES, InChi, ChemSpider, BioCyc, Metlin, PubChem  (Already in the previous model: BiGG, KEGG) |
| **Additional data included** | Metabolites monoisotopic mass and structures (SMILES),  Protein sequences |
| **Metabolic pathways added** | Coelimycin Biosynthesis  Butyrolactones Biosynthesis  Xylan Degradation  Cellulose Degradation |
| **Metabolic pathways updated** | Futalosine Pathway  Chitin Degradation  Oxidative Phosphorylation  NADH dehydrogenases  Cytochromes oxidases  Menaquinone utilization  Biomass  GAM/NGAM  Composition (menaquinones,  demethylmenaquinones, polyphosphate) |

**Table S4. Table of the new reactions added to the iAA1259 model.**

| **Reaction ID** | | **Reaction Name** | | | **Reaction Equation** | | | | | | **Genes Associated** |
| --- | --- | --- | --- | --- | --- | --- | --- | --- | --- | --- | --- |
| **CPKS1** | | CpkA initiation | | | ACPcpk[c] + 3 malcoa[c] + 3 h[c] <=> 3 coa[c] + hex24dACPcpk[c] + 3 co2[c] + 2 h2o[c] | | | | | | SCO6275 |
| **CPKS2** | | CpkB polyketide elongation 1 | | | hex24dACPcpk[c] + 2 malcoa[c] + 2 h[c] -> 2 coa[c] + h246dectACPcpk[c] + 2 co2[c] + h2o[c] | | | | | | SCO6274 |
| **CPKS3** | | CpkC polyketide elongation 2 and thioester reductase | | | h246dectACPcpk[c] + malcoa[c] + nadh[c] -> h2o[c] + hdd2610t[c] + 2 h[c] + co2[c] + nad[c] +coa[c]+ ACPcpk[c] | | | | | | SCO6273 |
| **CPKS4a** | | CpkG transaminase (with L-Ala) | | | hdd2610t[c] + ala-L[c] <=> 1add26810t5o[c] + pyr[c] | | | | | | SCO6279 |
| **CPKS4b** | | CpkG transaminase (with L-Glu) | | | hdd2610t[c] + glu-L[c] <=> 1add26810t5o[c] + akg[c] | | | | | | SCO6279 |
| **CPKS5** | | Flavin dependent epoxidases/dehydrogenases | | | add26810t5o[c] + fadh2[c] + 2 o2[c] -> cpkepox[c] + fad[c] 3 h2o[c] | | | | | | (SCO6276 or SCO6281 or SCO6272) |
| **CPKSt** | | Putative transmembrane efflux protein CpkF | | | cpkepox[c] -> cpkepox[e] | | | | | | SCO6278 |
| **CPKS6** | | Extracellular spontaneous reaction | | | cpkepox[e] + accyst[e] -> ycpk[e] + h2o[e] + 2 h[e] | | | | | | s0001 |
| **ACCS** | | N-acetylcysteine synthase (putative acyl-transferase) | | | accoa[c] + cys-L[c] -> accys[c] + coa[c] + h[c] | | | | | |  |
| **ACCt** | | N-acetylcysteine transport | | | accys[c] <=> accys[e] | | | | | |  |
| **ACPScpk** | | acyl-carrier protein synthase (cpk) | | | coa[c] + apoACPcpk[c] -> h[c] + ACPcpk[c] + pap[c] | | | | | | SCO4744 |
| **ACPSpdscpk** | | acyl-carrier-protein phosphodiesterase (cpk) | | | ACPcpk[c] + h2o[c] -> apoACPcpk[c] + pan4p[c] + h[c] | | | | | | SCO0046 |
| **XYLAN_DEGe** | | Xylan degradation (extracellular) | | | xylan[e] + 527 h2o[e] -> 528 xyl_D[e] | | | | | | (SCO5931 or SCO2292 or SCO1883 or SCO0674 or SCO0105) |
| **EX_xylan(e)** | | Xylan exchange | | | xylan[e] <=> | | | | | |  |
| **CELLUL_DEGe** | | Cellulose degradation (extracellular) | | | cellul[e] + 499 h2o[e] -> 250 celb[e] | | | | | | SCO6548 |
| **EX_cellul(e)** | | Cellulose exchange | | | cellul[e] <=> | | | | | |  |
| **CHTNDG** | | Chitin degradation | chitin[e] + h2o[e] -> acgam[e] + h[e] | | | | | (SCO7263 or SCO0482 or SCO1429 or SCO1444 or SCO5376 or SCO5954 or SCO6345 or SCO5003 or SCO5673 or SCO2833 or SCO6012 or SCO2503 or SCO7225) | | | |
| **CHDHR** | | Chorismate dehydratase | | chor[c] -> 3cvobz[c] + h2o[c] | | | | | | | SCO4506 |
| **ADXFUTSNT** | | Aminodeoxyfutalosine synthase | | 3cvobz[c] + amet[c] + h2o[c] -> 6adxfut[c] + met-L[c] + hco3[c] | | | | | | | SCO4494 |
| **ADXFUTDA** | | Aminodeoxyfutalosine deaminase | | 6adxfut[c] + h2o[c] -> fut[c] + nh4[c] | | | | | | | SCO5662 |
| **ADXFUTNS** | | Aminodeoxyfutalosine nucleosidase | | 6adxfut[c] + h2o[c] -> dhxfut[c] + ade[c] + h[c] | | | | | | |  |
| **FUTH** | | Futalosine hydrolase | | fut[c] + h2o[c] -> dhxfut[c] + hxan[c] | | | | | | | SCO4327 |
| **DXFUTOR** | | Dehypoxanthine futalosine:S-adenosyl-L-methionine oxidoreductase | | dhxfut[c] + amet[c] -> cdhxfut[c] + met-L[c] + dad-5[c] + h[c] | | | | | | | SCO4550 |
| **DH6NPHS** | | 1,4-dihydroxy-6-naphthoate synthase | | cdhxfut[c] -> dh6na[c] + glyald[c] | | | | | | | SCO4326 |
| **DHNAI** | | 1,4-dihydroxynaphthoate isomerization (hypothetical reaction) | | dh6na[c] -> dhna[c] | | | | | | |  |
| **DHNANT4** | | 1,4-dihydroxy-2-naphthoate nonaprenyltransferase | | dhna[c] + h[c] + nndp[c] -> 2dmmql9[c] + co2[c] + ppi[c] | | | | | | | (SCO4491 and/or SCO4556) |
| **SCB1_1** | | Acyl-transferase (using beta keto-acyl ACP precursor) | | dhap[c] + 3oiC9ACP[c] -> apoACP[c] + 2o3pp8m3onn[c] | | | | | | | SCO6266 |
| **SCB1_2** | | Aldol condensation SCB1 precursor (infered as spontaneous) | | 2o3pp8m3onn[c] -> h2o[c] + mh5odhf3mdp[c] | | | | | | | s0001 |
| **SCB1_3** | | Butenolide phosphatase reductase (SCB1 precursor) | | mh5odhf3mdp[c] + nadph[c] + h[c] -> mh5othfmdp[c] + nadp[c] | | | | | | | SCO6267 |
| **SCB1_4** | | SCB1 precursor phosphatase | | mh5othfmdp[c] + h2o[c] -> afactor[c] + pi[c] | | | | | | |  |
| **SCB1_5** | | Beta-keto-acid-CoA/ACP reductase | | afactor[c] + nadph{c] + h[c] -> scb1[c] + nadp[c] | | | | | | | SCO6264 |
| **SCB1t** | | SCB1 transport (diffusible molecule) | | scb1[c] <=> scb1[e] | | | | | | | s0001 |
| **SCB2_1a** | | Acyl-transferase (using beta keto-acyl CoA precursor) | | dhap[c] + 3odcoa[c] -> coa[c] + 2o3pop3odn[c] | | | | | | | SCO6266 |
| **SCB2_1b** | | Acyl-transferase (using beta keto-acyl ACP precursor) | | dhap[c] + 3oddecACP[c] -> apoACP[c] + 2o3pop3odn[c] | | | | | | | SCO6266 |
| **SCB2_2** | | Aldol condensation SCB2 precursor (infered as spontaneous) | | 2o3pop3odn[c]-> h2o[c] + 4o5odhmdhp[c] | | | | | | | s0001 |
| **SCB2_3** | | Butenolide phosphatase reductase (SCB2 precursor) | | 4o5odhmdhp[c] + nadph[c] + h[c] -> 4o5othf3mdp[c] + nadp[c] | | | | | | | SCO6267 |
| **SCB2_4** | | SCB2 precursor phosphatase | | 4o5othf3mdp[c] + h2o[c] -> 4hm3odhf2o[c] + pi[c] | | | | | | |  |
| **SCB2_5** | | Beta-keto-acid-CoA/ACP reductase | | 4hm3odhf2o[c] + nadph{c] + h[c] -> scb2[c] + nadp[c] | | | | | | | SCO6264 |
| **SCB2t** | | SCB2 transport (diffusible molecule) | | scb2[c] <=> scb2[e] | | | | | | | s0001 |
| **SCB3_1** | | Acyl-transferase (using beta keto-acyl ACP precursor) | | dhap[c] + 3oiC10ACP[c] -> apoACP[c] + 2o3pp8m3onn[c] | | | | | | | SCO6266 |
| **SCB3_2** | | Aldol condensation SCB3 precursor (infered as spontaneous) | | 2o3pp8m3onn[c] -> h2o[c] + 46mo5o25dhf3mdhp[c] | | | | | | | s0001 |
| **SCB3_3** | | Butenolide phosphatase reductase (SCB3 precursor) | | 46mo5o25dhf3mdhp[c] + nadph[c] + h[c] -> 465mo5othf3mdhp[c] + nadp[c] | | | | | | | SCO6267 |
| **SCB3_4** | | SCB3 precursor phosphatase | | 465mo5othf3mdhp[c] + h2o[c] -> 4hm36modhf2o[c] + pi[c] | | | | | | |  |
| **SCB3_5** | | Beta-keto-acid-CoA/ACP reductase | | 4hm36modhf2o[c]+ nadph{c] + h[c] -> scb3[c] + nadp[c] | | | | | | | SCO6264 |
| **SCB3t** | | SCB3 transport (diffusible molecule) | | scb3[c] <=> scb3[e] | | | | | | | s0001 |
| **CYOO** | | cytochrome o oxidase (menaquinol-9: 2 protons) | | 4 h[c] + mql9[c] + 0.5 o2[c] -> h2o[c] +mqn9[c] + 2h[e] | | | | | (SCO7234 and SCO7235 and SCO7236 and SCO1934 and SCO7120) | | |
| **NADH8** | | NADH dehydrogenase (demethylmenaquinone-9 & 3 protons) | | 2dmmq9[c] + 4 h[c] + nadh[c] -> 2dmmql9[c] + 3 h[e] + nad[c] | | | | | ((SCO4562 or SCO4599) and (SCO4563 or SCO4600) and SCO4564 and (SCO3392 or SCO4565) and SCO4566 and (SCO4567 or SCO6560) and SCO4568 and (SCO4569 or SCO4602) and (SCO4570 or SCO4603) and (SCO4571 or SCO4604) and (SCO4572 or SCO4605) and (SCO4573 or SCO4606 or SCO6954) and (SCO4574 or SCO4607) and (SCO4575 or SCO4608 or SCO6956)) | | |
| **NADH9** | | NADH dehydrogenase (demethylmenaquinone-9 & 0 protons) | | 2dmmq9[c] + h[c] + nadh[c] -> 2dmmql9[c] + nad[c] | | | | | (SCO3092 or SCO7101 or SCO7319 or SCO6496 or SCO0158 or SCO4119) | | |
| **NADPHQR4** | NADPH Quinone Reductase (2-Demethylmenaquinone-8) | | | | | | 2dmmq9[c] + h[c] + nadph[c] -> 2dmmql9[c] + nadp[c] | | | SCO3823 | |
| **AMMQT9r** | S-adenosylmethione:2-demthylmenaquinone methyltransferase (menaquinone 9) | | | | | 2dmmq9[c] + amet[c] <=> ahcys[c] + h[c] + mqn9[c] | | | | | (SCO4556 or SCO5940) |
| **FRD3** | fumarate reductase (2-demthylmenaquinone-9) | | | | | 2dmmql9[c] + fum[c] -> 2dmmq9c] + succ[c] | | | | | (SCO0923 and SCO0922 and SCO0924) |
| **G3PD7** | glycerol-3-phosphate dehydrogenase (2-demthylmenaquinone-9) | | | | | 2dmmq8[c] + glyc3p[c] -> 2dmmql8[c] + dhap[c] | | | | | (SCO0670 or SCO1661 or SCO4774 or (SCO7005 and SCO7006)) |
| **GLYCTO4** | Glycolate oxidase | | | | | 2dmmq8[c] + glyclt[c] -> 2dmmql8[c] + glx[c] | | | | | SCO2925 |

Table S5. Table of the new metabolites added to the iAA1259 model

| **Metabolite name** | **Metabolite description** | **Neutral metabolite formula** | **Metabolite Compartment** |
| --- | --- | --- | --- |
| **2o3pop3odn[c]** | 2-oxo-3-(phosphonooxy)propyl 3-oxodecanoate | C13H23O8P | Cytosol |
| **2o3popp8m3odn[c]** | 2-oxo-3-(phosphonooxy)propyl 8-methyl-3-oxodecanoate | C14H25O8P | Cytosol |
| **2o3pp8m3onn[c]** | 2-oxo-3-(phosphonooxy)propyl 8-methyl-3-oxononanoate | C13H23O8P | Cytosol |
| **3cvobz[c]** | 3-[(1-Carboxyvinyl)oxy]benzoate | C10H6O5 | Cytosol |
| **465mo5othf3mdhp[c]** | (3S,4S)-4-(6-methyloctanoyl)-5-oxotetrahydrofuran-3-yl)methyl dihydrogen phosphate | C14H25O7P | Cytosol |
| **46mo5o25dhf3mdhp[c]** | (4-(6-methyloctanoyl)-5-oxo-2,5-dihydrofuran-3-yl)methyl dihydrogen phosphate | C14H23O7P | Cytosol |
| **4hm36modhf2o[c]** | (3S,4R)-4-(hydroxymethyl)-3-(6-methyloctanoyl)dihydrofuran-2(3H)-one | C14H24O4 | Cytosol |
| **4hm3odhf2o[c]** | (3S,4R)-4-(hydroxymethyl)-3-octanoyldihydrofuran-2(3H)-one | C13H22O4 | Cytosol |
| **4o5odhmdhp[c]** | (4-octanoyl-5-oxo-2,5-dihydrofuran-3-yl)methyl dihydrogen phosphate | C13H21O7P | Cytosol |
| **4o5othf3mdp[c]** | ((3S,4S)-4-octanoyl-5-oxotetrahydrofuran-3-yl)methyl dihydrogen phosphate | C13H23O7P | Cytosol |
| **6adxfut[c]** | 6-Amino-6-deoxyfutalosine | C19H19N5O6 | Cytosol |
| **accys[c]** | N-Acetyl-L-Cysteine | C5H9NO3S | Cytosol |
| **accys[e]** | N-Acetyl-L-Cysteine | C5H9NO3S | Extra-organism |
| **ACPcpk[c]** | Acyl carrier protein (specific to actinorhodin coelimycin) | C11H22N2O7PRS | Cytosol |
| **add26810t5o[c]** | 1-Aminododeca-2,6,8,10-tetraen-5-ol | C12H19NO | Cytosol |
| **afactor[c]** | A-factor: (4R)-4-(hydroxymethyl)-3-(6-methylheptanoyl)oxolan-2-one | C13H22O4 | Cytosol |
| **apoACPcpk[c]** | apoprotein [acyl carrier protein] (cpk) | RHO | Cytosol |
| **cdhxfut[c]** | Cyclic dehypoxanthine futalosine | C14H14O7 | Cytosol |
| **cellul[e]** | Cellulose, chain length 500 glc_D assumed, DOI: 10.1002/anie.200460587 | C3000H5002O2501 | Extra-organism |
| **chitin[e]** | Chitin monomer | C8H13NO5 | Extra-organism |
| **cpkepox[c]** | 6-(3'-(prop-1-en-1-yl)-[2,2'-bioxiran]-3-yl)-2,3-dihydropyridine | C12H15NO2 | Cytosol |
| **cpkepox[e]** | 6-(3'-(prop-1-en-1-yl)-[2,2'-bioxiran]-3-yl)-2,3-dihydropyridine | C12H15NO2 | Extra-organism |
| **h246dectACPcpk[c]** | 3-Hydroxy-2,4,6-Decatetraenoyl-[acyl-carrier protein] | C21H34N2O9PRS | Cytosol |
| **hdd2610t[c]** | 5-Hydroxydodeca-2,6,8,10-tetraenal | C12H16O2 | Cytosol |
| **hex24dACPcpk[c]** | 2,4-Hexadienoyl-ACP (n-C6:2ACP) | C17H30N2O8PRS | Cytosol |
| **mh5odhfmdp[c]** | (4-(6-methylheptanoyl)-5-oxo-2,5-dihydrofuran-3-yl)methyl dihydrogen phosphate | C13H21O7P | Cytosol |
| **mh5othfmdp[c]** | ((3S,4S)-4-(6-methylheptanoyl)-5-oxotetrahydrofuran-3-yl)methyl dihydrogen phosphate | C13H23O7P | Cytosol |
| **scb1[c]** | SCB1: (3S,4R)-3-((R)-1-hydroxy-6-methylheptyl)-4-(hydroxymethyl)dihydrofuran-2(3H)-one | C13H24O4 | Cytosol |
| **scb1[e]** | SCB1: (3S,4R)-3-((R)-1-hydroxy-6-methylheptyl)-4-(hydroxymethyl)dihydrofuran-2(3H)-one | C13H24O4 | Extra-organism |
| **scb2[c]** | SCB2: (3S,4R)-4-(hydroxymethyl)-3-((R)-1-hydroxyoctyl)dihydrofuran-2(3H)-one | C13H24O4 | Cytosol |
| **scb2[e]** | SCB2: (3S,4R)-4-(hydroxymethyl)-3-((R)-1-hydroxyoctyl)dihydrofuran-2(3H)-one | C13H24O5 | Extra-organism |
| **scb3[c]** | SCB3: (3S,4R)-3-((1R)-1-hydroxy-6-methyloctyl)-4-(hydroxymethyl)dihydrofuran-2(3H)-one | C14H26O4 | Cytosol |
| **scb3[e]** | SCB3:(3S,4R)-3-((1R)-1-hydroxy-6-methyloctyl)-4-(hydroxymethyl)dihydrofuran-2(3H)-one | C14H26O4 | Extra-organism |
| **xylan[e]** | Oat spelt xylan, MW 79200 (DOI: 10.1002/masy.200551405) = 528 xyl_D | C2640H4226O2113 | Extra-organism |

Table S6. Table of the new genes added to the iAA1259 model

| **Gene** | **Type of Addition** | **Characterization Year** | **Reference** |
| --- | --- | --- | --- |
| **SCO0105** | new content added | 2016 | Enkhbaatar, Bolormaa, et al. "Molecular characterization of xylobiose-and xylopentaose-producing β-1, 4-endoxylanase SCO5931 from Streptomyces coelicolor A3 (2)." Applied biochemistry and biotechnology 180.2 (2016): 349-360. |
| **SCO0284** | update of GPR |  | KEGG (alpha-galactosidase) |
| **SCO0382** | update of GPR |  | KEGG (UDPglucose dehydrogenase) |
| **SCO0462** | update of GPR |  | KEGG (2-dehydropantoate 2-reductase) |
| **SCO0482** | new content added |  | BioCyc (chitinase) |
| **SCO0554** | update of GPR |  | KEGG (endoglucanase) |
| **SCO0674** | new content added | 2016 | Enkhbaatar, Bolormaa, et al. "Molecular characterization of xylobiose-and xylopentaose-producing β-1, 4-endoxylanase SCO5931 from Streptomyces coelicolor A3 (2)." Applied biochemistry and biotechnology 180.2 (2016): 349-360. |
| **SCO0765** | update of GPR |  | KEGG (endoglucanase) |
| **SCO0984** | update of GPR |  | KEGG ( 3-hydroxyacyl-CoA dehydrogenase) |
| **SCO1268** | update of GPR |  | KEGG (Dihydrolipoamide acetyltransferase component of pyruvate dehydrogenase complex)- PDH |
| **SCO1429** | new content added |  | KEGG (chitinase) |
| **SCO1444** | new content added | 2013 | KEGG (chitinase) + Nazari, Behnam, et al. "Chitin-induced gene expression in secondary metabolic pathways of Streptomyces coelicolor A3 (2) grown in soil." Applied and environmental microbiology 79.2 (2013): 707-713 |
| **SCO1883** | new content added | 2016 | Enkhbaatar, Bolormaa, et al. "Molecular characterization of xylobiose-and xylopentaose-producing β-1, 4-endoxylanase SCO5931 from Streptomyces coelicolor A3 (2)." Applied biochemistry and biotechnology 180.2 (2016): 349-360. |
| **SCO2154** | update of GPR | 2017 | Sawers, R. G., D. Falke, and M. Fischer. "Chapter One-Oxygen and Nitrate Respiration in Streptomyces coelicolor A3 (2)." *Advances in microbial physiology* 68 (2016): 1-40. |
| **SCO2292** | new content added | 2016 | Enkhbaatar, Bolormaa, et al. "Molecular characterization of xylobiose-and xylopentaose-producing β-1, 4-endoxylanase SCO5931 from Streptomyces coelicolor A3 (2)." Applied biochemistry and biotechnology 180.2 (2016): 349-360. |
| **SCO2503** | new content added | 2013 | Nazari, Behnam, et al. "Chitin-induced gene expression in secondary metabolic pathways of Streptomyces coelicolor A3 (2) grown in soil." Applied and environmental microbiology 79.2 (2013): 707-717 |
| **SCO2833** | new content added | 2013 | Nazari, Behnam, et al. "Chitin-induced gene expression in secondary metabolic pathways of Streptomyces coelicolor A3 (2) grown in soil." Applied and environmental microbiology 79.2 (2013): 707-715 |
| **SCO3823** | new content added |  | Blast against SCO tr|A0A024H6J2|A0A024H6J2_9MICC NAD(P)H quinone oxidoreductase, PIG3 family protein OS=Pseudarthrobacter siccitolerans GN=qor PE=4 SV=1 |
| **SCO3835** | update of GPR | 2017 | Millan-Oropeza, Aaron, et al. "Quantitative proteomic analysis confirmed oxidative metabolism predominates in Streptomyces coelicolor versus glycolytic metabolism in Streptomyces lividans." Journal of Proteome Research (2017). |
| **SCO4326** | new content added | 2013 | Mahanta, Nilkamal, et al. "Menaquinone biosynthesis: formation of aminofutalosine requires a unique radical SAM enzyme." *Journal of the American Chemical Society* 135.41 (2013). |
| **SCO4327** | new content added | 2013 | Mahanta, Nilkamal, et al. "Menaquinone biosynthesis: formation of aminofutalosine requires a unique radical SAM enzyme." *Journal of the American Chemical Society* 135.41 (2013). |
| **SCO4491** | update of GPR | 2013 | Cooper, Lisa E., et al. "In vitro reconstitution of the radical SAM enzyme MqnC involved in the biosynthesis of futalosine-derived menaquinone." Biochemistry 52.27 (2013) |
| **SCO4494** | new content added | 2013 | Mahanta, Nilkamal, et al. "Menaquinone biosynthesis: formation of aminofutalosine requires a unique radical SAM enzyme." *Journal of the American Chemical Society* 135.41 (2013). |
| **SCO4506** | new content added | 2013 | Mahanta, Nilkamal, et al. "Menaquinone biosynthesis: formation of aminofutalosine requires a unique radical SAM enzyme." *Journal of the American Chemical Society* 135.41 (2013). |
| **SCO4550** | new content added | 2013 | Mahanta, Nilkamal, et al. "Menaquinone biosynthesis: formation of aminofutalosine requires a unique radical SAM enzyme." *Journal of the American Chemical Society* 135.41 (2013). |
| **SCO4556** | update of GPR | 2013 | Cooper, Lisa E., et al. "In vitro reconstitution of the radical SAM enzyme MqnC involved in the biosynthesis of futalosine-derived menaquinone." Biochemistry 52.27 (2013) |
| **SCO4655** | update of GPR | 2017 | Millan-Oropeza, Aaron, et al. "Quantitative proteomic analysis confirmed oxidative metabolism predominates in Streptomyces coelicolor versus glycolytic metabolism in Streptomyces lividans." Journal of Proteome Research (2017). |
| **SCO5003** | new content added | 2013 | Nazari, Behnam, et al. "Chitin-induced gene expression in secondary metabolic pathways of Streptomyces coelicolor A3 (2) grown in soil." Applied and environmental microbiology 79.2 (2013): 707-713 |
| **SCO5376** | new content added | 2013 | KEGG (chitinase) + Nazari, Behnam, et al. "Chitin-induced gene expression in secondary metabolic pathways of Streptomyces coelicolor A3 (2) grown in soil." Applied and environmental microbiology 79.2 (2013): 707-713 |
| **SCO5498** | update of GPR |  | KEGG (Glutamyl-tRNA synthetase) |
| **SCO5499** | update of GPR |  | KEGG (Glutamyl-tRNA synthetase) |
| **SCO5662** | new content added | 2013 | Mahanta, Nilkamal, et al. "Menaquinone biosynthesis: formation of aminofutalosine requires a unique radical SAM enzyme." *Journal of the American Chemical Society* 135.41 (2013). |
| **SCO5673** | new content added | 2013 | Nazari, Behnam, et al. "Chitin-induced gene expression in secondary metabolic pathways of Streptomyces coelicolor A3 (2) grown in soil." Applied and environmental microbiology 79.2 (2013): 707-714 |
| **SCO5931** | new content added | 2016 | Enkhbaatar, Bolormaa, et al. "Molecular characterization of xylobiose-and xylopentaose-producing β-1, 4-endoxylanase SCO5931 from Streptomyces coelicolor A3 (2)." Applied biochemistry and biotechnology 180.2 (2016): 349-360. |
| **SCO5954** | new content added |  | KEGG (chitinase) |
| **SCO6012** | new content added | 2013 | Nazari, Behnam, et al. "Chitin-induced gene expression in secondary metabolic pathways of Streptomyces coelicolor A3 (2) grown in soil." Applied and environmental microbiology 79.2 (2013): 707-716 |
| **SCO6272** | new content added | 2012 | Gomez-Escribano JP, Song L, Fox DJ, Yeo V, Bibb MJ, Challis GL. Structure and biosynthesis of the unusual polyketide alkaloid coelimycin P1, a metabolic product of the cpk gene cluster of Streptomyces coelicolor M145. Chemical Science. 2012;3(9):2716-20. |
| **SCO6273** | new content added | 2017 | Awodi UR, Ronan JL, Masschelein J, de los Santos EL, Challis GL. Thioester reduction and aldehyde transamination are universal steps in actinobacterial polyketide alkaloid biosynthesis. Chemical Science. 2017;8(1):411-5. Pawlik K, Kotowska M, Chater KF, Kuczek K, Takano E. A cryptic type I polyketide synthase (cpk) gene cluster in Streptomyces coelicolor A3 (2). Archives of microbiology. 2007 Feb 1;187(2):87-99. |
| **SCO6274** | new content added | 2007 | Pawlik K, Kotowska M, Chater KF, Kuczek K, Takano E. A cryptic type I polyketide synthase (cpk) gene cluster in Streptomyces coelicolor A3 (2). Archives of microbiology. 2007 Feb 1;187(2):87-99. |
| **SCO6275** | new content added | 2007 | Pawlik K, Kotowska M, Chater KF, Kuczek K, Takano E. A cryptic type I polyketide synthase (cpk) gene cluster in Streptomyces coelicolor A3 (2). Archives of microbiology. 2007 Feb 1;187(2):87-99. |
| **SCO6276** | new content added | 2012 | Gomez-Escribano JP, Song L, Fox DJ, Yeo V, Bibb MJ, Challis GL. Structure and biosynthesis of the unusual polyketide alkaloid coelimycin P1, a metabolic product of the cpk gene cluster of Streptomyces coelicolor M145. Chemical Science. 2012;3(9):2716-20. |
| **SCO6278** | new content added | 2012 | Gomez-Escribano JP, Song L, Fox DJ, Yeo V, Bibb MJ, Challis GL. Structure and biosynthesis of the unusual polyketide alkaloid coelimycin P1, a metabolic product of the cpk gene cluster of Streptomyces coelicolor M145. Chemical Science. 2012;3(9):2716-20. |
| **SCO6279** | new content added | 2017 | Awodi UR, Ronan JL, Masschelein J, de los Santos EL, Challis GL. Thioester reduction and aldehyde transamination are universal steps in actinobacterial polyketide alkaloid biosynthesis. Chemical Science. 2017;8(1):411-5. Pawlik K, Kotowska M, Chater KF, Kuczek K, Takano E. A cryptic type I polyketide synthase (cpk) gene cluster in Streptomyces coelicolor A3 (2). Archives of microbiology. 2007 Feb 1;187(2):87-99. |
| **SCO6279** | new content added | 2017 | Awodi UR, Ronan JL, Masschelein J, de los Santos EL, Challis GL. Thioester reduction and aldehyde transamination are universal steps in actinobacterial polyketide alkaloid biosynthesis. Chemical Science. 2017;8(1):411-5. Pawlik K, Kotowska M, Chater KF, Kuczek K, Takano E. A cryptic type I polyketide synthase (cpk) gene cluster in Streptomyces coelicolor A3 (2). Archives of microbiology. 2007 Feb 1;187(2):87-99. |
| **SCO6281** | new content added | 2012 | Gomez-Escribano JP, Song L, Fox DJ, Yeo V, Bibb MJ, Challis GL. Structure and biosynthesis of the unusual polyketide alkaloid coelimycin P1, a metabolic product of the cpk gene cluster of Streptomyces coelicolor M145. Chemical Science. 2012;3(9):2716-20. |
| **SCO6345** | new content added |  | BioCyc (chitinase) |
| **SCO6548** | new content added | 2016 | Lim, Ju-Hyeon, et al. "Molecular characterization of Streptomyces coelicolor A (3) SCO6548 as a cellulose 1, 4-β-cellobiosidase." FEMS microbiology letters 363.3 (2016) |
| **SCO6712** | misannotation | 2013 | Sherif, Mohammed, et al. "Biochemical studies of the multicopper oxidase (small laccase) from Streptomyces coelicolor using bioactive phytochemicals and site‐directed mutagenesis." *Microbial biotechnology* 6.5 (2013): 588-597 |
| **SCO6956** | new content added | 2017 | Sawers, R. G., D. Falke, and M. Fischer. "Chapter One-Oxygen and Nitrate Respiration in Streptomyces coelicolor A3 (2)." *Advances in microbial physiology* 68 (2016): 1-40. |
| **SCO7225** | new content added | 2013 | Nazari, Behnam, et al. "Chitin-induced gene expression in secondary metabolic pathways of Streptomyces coelicolor A3 (2) grown in soil." Applied and environmental microbiology 79.2 (2013): 707-718 |
| **SCO7263** | new content added | 2013 | BioCyc (chitinase) + Nazari, Behnam, et al. "Chitin-induced gene expression in secondary metabolic pathways of Streptomyces coelicolor A3 (2) grown in soil." Applied and environmental microbiology 79.2 (2013): 707-713 |
| **SCO7266** | update of GPR |  | KEGG (3-oxoacyl-[acyl-carrier-protein] reductase)- PDH |
| **SCO7637** | update of GPR |  | KEGG (endoglucanase) |

1. **Correlation analysis between gene expression and predicted fluxes for iMA789 and iMK1208 (gene expression showing a variation superior to 25%)**


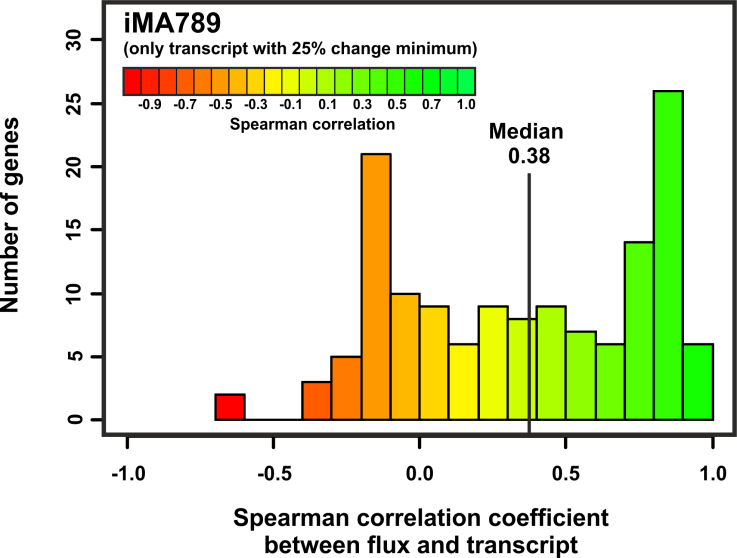

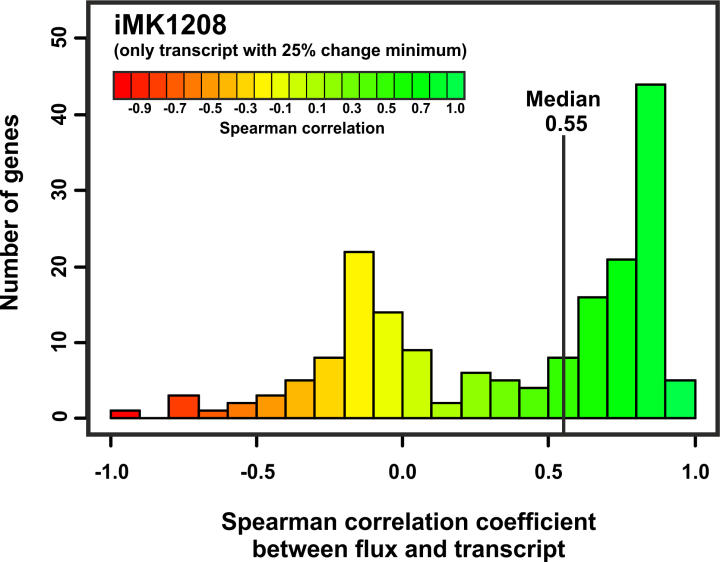


**Fig. S1. Correlation analysis of gene expression to predicted fluxes of iMA789, and iMK1208 (for transcripts showing a change in level of expression superior to 25%).**

a) Histogram of correlations for the iMA789 model, only taking in account genes with expression variation of more than 25% between the minimal and maximal transcript level.

b) Histogram of correlations for the iMK1208 model, only taking in account genes with expression variation of more than 25% between the minimal and maximal transcript level.

For both cases there is an increase of correlation compared to the whole expression dataset correlation, iMA789 goes from an overall Pearson correlation of 0.13 to 0.38, and iMK1208 from 0.18 to 0.55. This emphasis the improvements in predictions showed by iAA1259, with an increase in correlation from 0.56 to 0.78 when filtering for changing transcripts (Fig. 3c and 3d).

1. **Automated mapping of an untargeted metabolomics dataset onto the metabolic network iAA1259, using standardized metabolite identifiers**


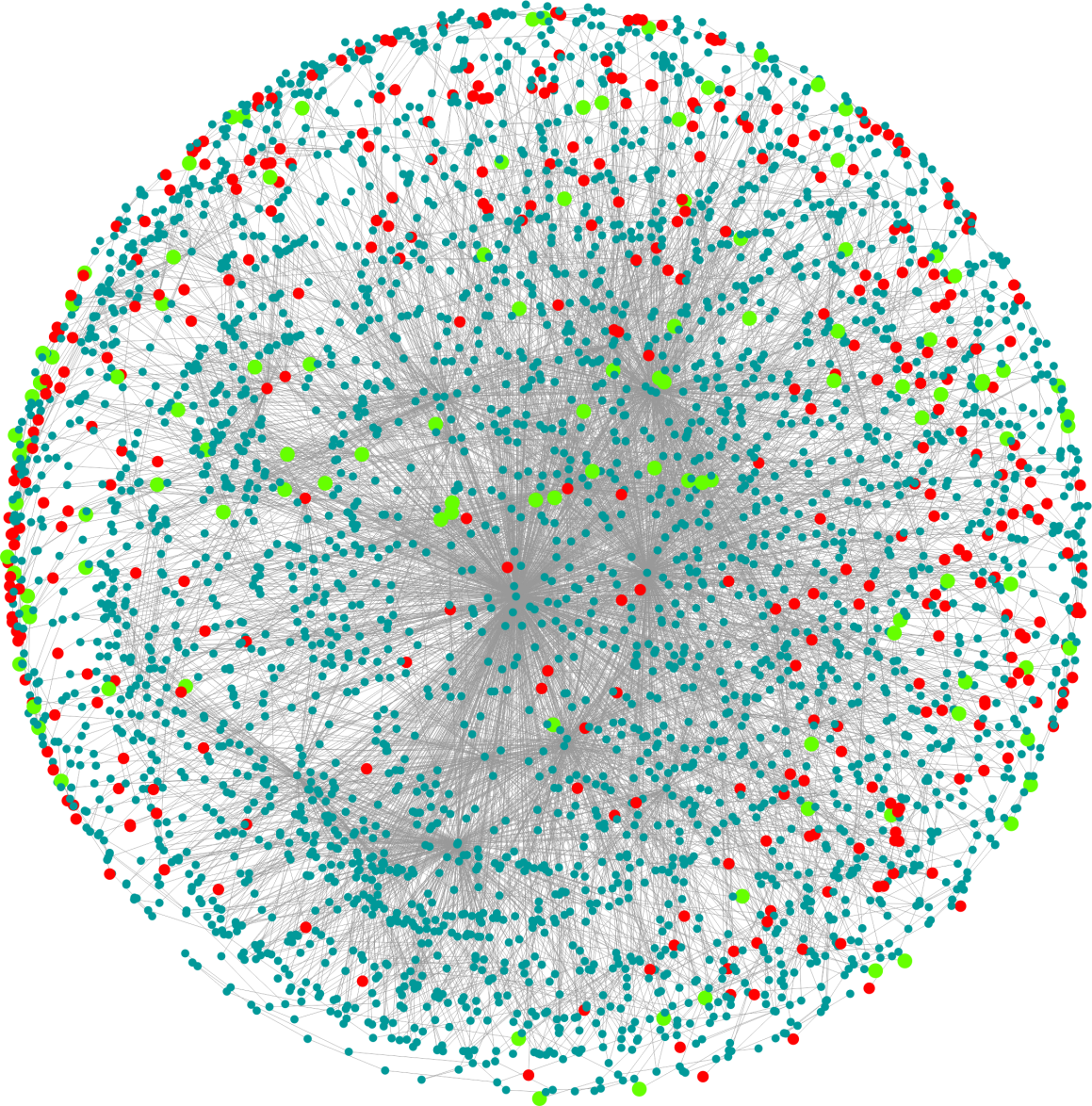


Fig. S2. Mapping of observed metabolites in an untargeted metabolomics dataset onto the metabolic network. To illustrate the easy integration of experimental data with the computational model, once standard metabolite identifiers are used, an LC-MS metabolomics dataset obtained from the literature (Jankevics et al., 2011) was automatically processed and annotated using MzMatch, then mapped onto the updated metabolic network iAA1259. The red dots correspond to the metabolites putatively annotated in the dataset (level 2 of confidence for metabolite identification [1]) , and the green dots represent the metabolites annotated with high confidence using chemical standards (level 1 of confidence for metabolite identification [1]). Figure generated with the software Cytoscape (Shannon et al., 2003). Once metabolites have been mapped, flux estimates inferred from the metabolomics experiment can, e.g., be used as additional flux constraints. A similar analysis based on traditional models using arbitrary metabolite identifiers would require manual curation for each metabolite and would rapidly become prohibitively time-consuming.

**Supplementary Method for the metabolomics data analysis and mapping**

The untargeted metabolomics dataset used for illustration of the metabolite mapping enabled by the standard identifiers used in the updated model is derived from a previous *S. coelicolor* study using LC-MS (HILIC and C18 columns), with a large number of technical and biological replicates [2]. The data was processed and analysed using MzMatch [3], using a similar protocol as described in the initial study [2]; however, only metabolites putatively annotated in at least 80 samples were retained for the visualization. The annotated metabolites were automatically mapped to their corresponding annotation in the model, which was used as an input for highlighting the detected metabolites on the metabolic network visualisation in Cytoscape [4].

1. **Constraints and *in-silico* growth rates predictions used for the Fig. 1**

Table S7. Constraints used and predicted growth rates of the different models

| **Sample N°** | **Glucose uptake**  **(mmol/gDW.h)** | **O_2_**  **(mmol/g.h)** | **CO_2_ (mmol/g.h)** | **Actinorhodin**  **(μ g/g.h)** |
| --- | --- | --- | --- | --- |
| **1** | 0.5 | 1.8 | 1.9 | 2 |
| **2** | 0.6 | 2 | 2 | 2 |
| **3** | 0.8 | 2.4 | 2.5 | 415 |
| **4** | 0.9 | 2.5 | 2.7 | 152 |
| **5** | 1.1 | 3.1 | 3.1 | 60 |
| **6** | 1.85 | 6.6 | 6.7 | 7 |
| **7** | 2.1 | 7.2 | 7 | 5 |

| **Sample N°** | **Measured Growth Rate**  **(gDW/h)** | **Predicted Growth Rate iIB711 (gDW/h)** | **Predicted Growth Rate iMA789**  **(gDW/h)** | **Predicted Growth Rate iMK1208**  **(gDW/h)** | **Predicted Growth Rate iAA1259**  **(gDW/h)** |
| --- | --- | --- | --- | --- | --- |
| **1** | 0.035 | 0.0185 | 0.0272 | 0.0278 | 0.0284 |
| **2** | 0.045 | 0.0253 | 0.0396 | 0.0405 | 0.0412 |
| **3** | 0.06 | 0.0382 | 0.0539 | 0.0577 | 0.0588 |
| **4** | 0.072 | 0.0414 | 0.0657 | 0.0681 | 0.0694 |
| **5** | 0.092 | 0.0615 | 0.0862 | 0.0885 | 0.0901 |
| **6** | 0.115 | 0.1770 | 0.1088 | 0.1113 | 0.1134 |
| **7** | 0.128 | 0.1965 | 0.1385 | 0.1417 | 0.1443 |

This table contains the data used to constrain the genome-scale metabolic models (top table), these data were acquired by Melzoch et al. [5]. The table also contains the experimental specific growth rates, and the growth rates predicted by the different metabolic models of *S. coelicolor* (bottom part): iIB711 [6], iMA789 [7], iMK1209 [8], and iAA1259.

1. **Metabolic models qualitative biomass predictions comparison**

**
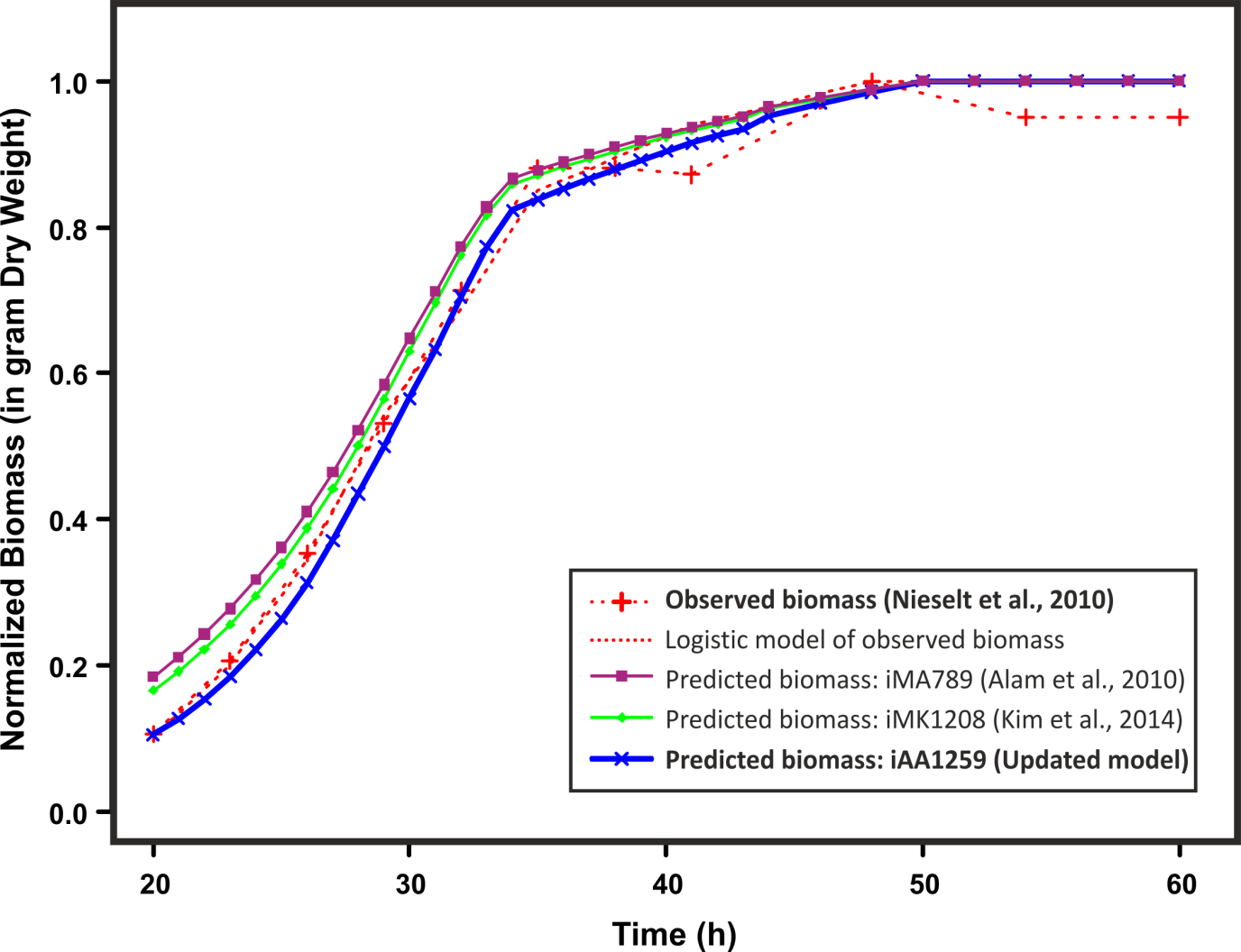
**

Fig. S3. Comparison of the normalized growth prediction of the metabolic models to the experimental data

When the dynamic growth data is normalized, the iAA1259 model shows a qualitative improvement in the prediction of the biomass. The iAA1259 model predictions (purple curve) are closer to the experimental data (red curve) than the previous models iMA789 (pink) and iMK1208 (green). The iAA1259 seems to enable better prediction of the growth curve from exponential to stationary phase.

**Supplementary References**

1. Sansone S-A, Fan T, Goodacre R, Griffin JL, Hardy NW, Kaddurah-Daouk R, et al. The Metabolomics Standards Initiative. Nat. Biotechnol. 2007;25:846–8.

2. Jankevics A, Merlo ME, de Vries M, Vonk RJ, Takano E, Breitling R. Metabolomic analysis of a synthetic metabolic switch in *Streptomyces coelicolor* A3 (2). Proteomics. 2011;11:4622–31.

3. Scheltema RA, Jankevics A, Jansen RC, Swertz MA, Breitling R. PeakML/mzMatch: a file format, Java library, R library, and tool-chain for mass spectrometry data analysis. Anal. Chem. 2011;83:2786–93.

4. Shannon P, Markiel A, Ozier O, Baliga NS, Wang JT, Ramage D, et al. Cytoscape: a software environment for integrated models of biomolecular interaction networks. Genome Res. 2003;13:2498–504.

5. Melzoch K, De Mattos MJT, Neijssel OM. Production of actinorhodin by *Streptomyces coelicolor* A3(2) grown in chemostat culture. Biotechnol. Bioeng. 1997;54:577–82.

6. Borodina I, Krabben P, Nielsen J. Genome-scale analysis of *Streptomyces coelicolor* A3(2) metabolism. Genome Res. 2005;15:820–9.

7. Alam MT, Merlo ME, Hodgson DA, Wellington EMH, Takano E, Breitling R. Metabolic modeling and analysis of the metabolic switch in *Streptomyces coelicolor*. BMC Genomics. 2010;11:1.

8. Kim M, Yi JS, Kim J, Kim JN, Kim MW, Kim BG. Reconstruction of a high-quality metabolic model enables the identification of gene overexpression targets for enhanced antibiotic production in *Streptomyces coelicolor* A3(2). Biotechnol. J. 2014;9:1185–94.
