# Supplementary figures and images for: Development and validation of an updated computational model of Streptomyces coelicolor primary and secondary metabolism
Source: BMC Genomics. 2018 Jul 4;19:519. doi: 10.1186/s12864-018-4905-5 (PMC6040156; doi:10.1186/s12864-018-4905-5)

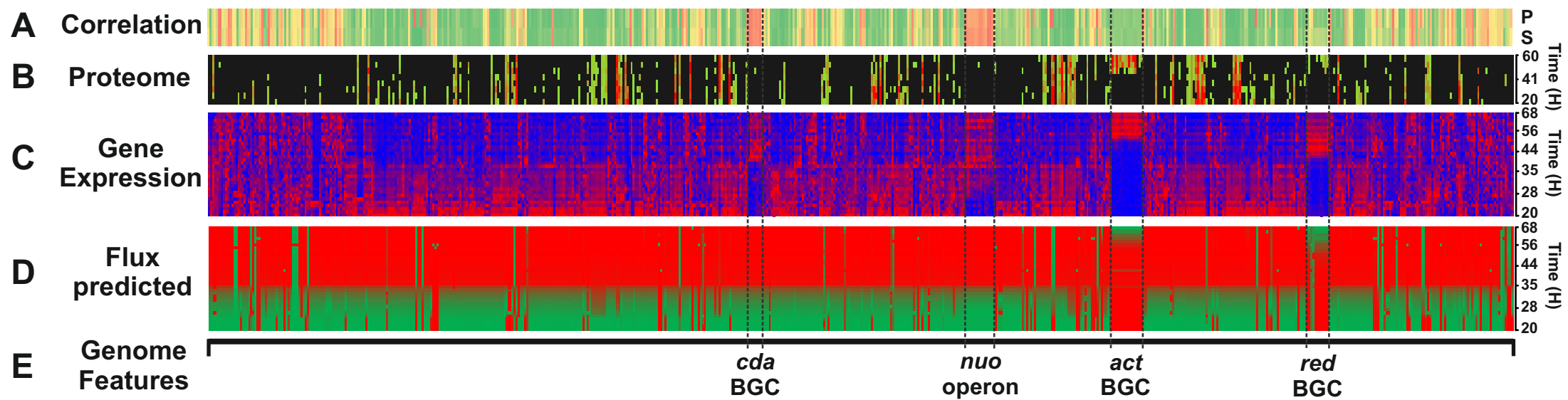

Supplement: Supplementary file 5 — High-resolution version of Fig. 4. Validation by integrated transcriptomics and proteomics analysis. (PDF 1881 kb) [file 12864_2018_4905_MOESM5_ESM.pdf]
